# Single-cell genomics reveals new rozellid lineages and supports their sister relationship to Microsporidia

**DOI:** 10.1098/rsbl.2023.0398

**Published:** 2023-12-13

**Authors:** Pauline C. Thomé, Iker Irisarri, Justyna Wolinska, Michael T. Monaghan, Jürgen F. H. Strassert

**Affiliations:** ^1^ Department of Evolutionary and Integrative Ecology, Leibniz Institute of Freshwater Ecology and Inland Fisheries (IGB), Berlin, Germany; ^2^ Section Phylogenomics, Centre for Molecular Biodiversity Research, Leibniz Institute for the Analysis of Biodiversity Change, Museum of Nature Hamburg, Hamburg, Germany; ^3^ Institut für Biologie, Freie Universität Berlin, Berlin, Germany

**Keywords:** Rozellomycota, Microsporidia, Holomycota, phylogenomics, mitochondrion, mitosome

## Abstract

The phylum Rozellomycota has been proposed for a group of early-branching holomycotan lineages representing obligate parasites and hyperparasites of zoosporic fungi, oomycotes or phytoplankton. Given their predominantly intracellular lifestyle, rozellids are typically known from environmental ribosomal DNA data, except for the well-studied *Rozella* species. To date, the phylogenetic relationship between rozellids and microsporidians (Microsporidia) is not fully understood and most reliable hypotheses are based on phylogenomic analyses that incorporate the only publicly available rozellid genome of *Rozella allomycis*. Here, we provide genomic data of three new rozellid lineages obtained by single-cell sequencing from environmental samples and show with a phylogenomic approach that rozellids form a monophyletic group that is sister to microsporidians, corroborating the previously proposed phylum Rozellomycota. Whereas no mitochondrial genes coding for the respiratory Complex I could be found, we discovered a gene coding for a nucleotide phosphate transporter in one of the three draft genomes. The scattered absence of Complex I genes and scattered presence of nucleotide transporter genes across diverse microsporidian and rozellid lineages suggest that these adaptations to a parasitic lifestyle, which reduce the parasite's capability to synthesize ATP but enables it to steal ATP from its host, evolved independently in microsporidians and rozellids.

## 1. Introduction

The early-branching holomycotan phylum Rozellomycota (rozellids) was proposed based on a few formally described species of the genus *Rozella* as well as on environmental sequences obtained by metabarcoding or by fluorescence *in situ* hybridizations (FISH) combined with single-cell sequencing [1,–7], initially named Cryptomycota [8]. Most of what is known about rozellids is deduced from the well-studied *Rozella* species, which are obligate parasites that disperse as flagellated zoospores and feed by phagocytosis as wall-less endobiotic amoeboids growing inside the host cell while adapting its cell wall and thereby shape to form sporangia or alternatively resting spores [5,9]. In *Rozella*, a reduced mitochondrion lacking Complex I was reported, causing a dependence on importing nucleotides from the hosts [10,11], which are often parasites themselves and belong to the zoosporic fungi Blastocladiomycota or Chytridiomycota (chytrids), or to the Oomycota (Stramenopila) [2,5]. Other rozellids and rozellid-associated lineages were suggested to parasitize phytoplankton and were shown by FISH to comprise both endo- and epiparasites [3,12–15]. Environmental and metabarcoding surveys showed that rozellids are ubiquitous in different climates and various freshwater, marine and soil habitats [3,4,6,7,16–19]. As parasites, they are hypothesized to influence host populations [13,20,21] and their zoospores may serve as a nutritious food source, as is known for other fungal zoospores [22,23].

Based on ribosomal DNA phylogenies, morphology and metabolic characteristics, the early-branching holomycotan phyla Aphelidiomycota (aphelids), Microsporidia (microsporidians) and Rozellomycota were established [1,24,25]. Yet, all these investigations have failed so far to infer their deep phylogenetic relationships with confidence [9,26–29]. Only when more genomic and transcriptomic data allowed for phylogenomic analyses, the putative rozellids *Paramicrosporidium saccamoebae* and *Mitosporidium daphniae* [29,30] were suggested to belong to the microsporidians [25,31,32], but the delineation remained controversial [33,34]. So did the position of the only safely assigned rozellid (*R. allomycis*) for which genomic data have been made publicly available to date and hence the relationship between microsporidians and rozellids in general [31,35]. Here, we present genomic data of three new rozellid lineages uncovered from environmental single-cell samples and confirm the previously hypothesized sister relationship of rozellids to microsporidians with full support throughout all phylogenomic analyses. We furthermore report on a nucleotide transporter protein found in one of the rozellid draft genomes and speculate on its possible function in adapting to a parasitic lifestyle.

## 2. Methods

**Figure 1.**
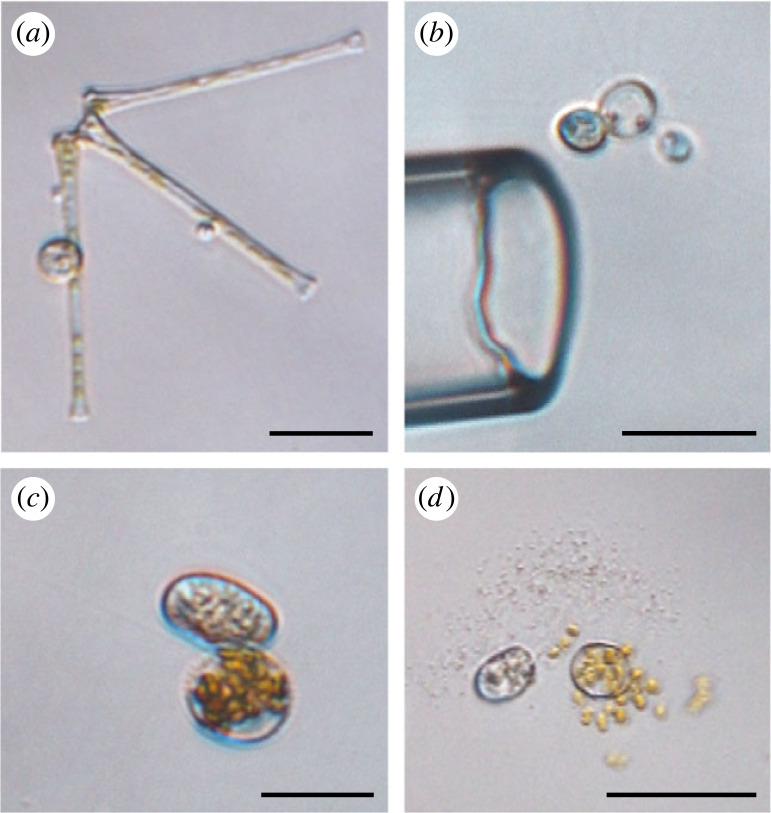
Parasites and their hosts from environmental samples. Identifications were based on morphological characteristics in combination with phylogenomic tree inference (see [36]) and with BLASTn and diamond BLASTx searches against the SILVA SSU database [37] and the NCBI nr database, respectively. Samples were collected from Lake Müggelsee (Berlin, Germany). Scale bars: 25 µm. (*a*) *Asterionella* with sporangium; contained Rozellid 19–20. (*b*) Putative *Golenkinia* with sporangium; contained Rozellid 133–135. (*c–d*) Centric diatom with sporangium; contained Rozellid 233–234 plus chytrid *Rhizophydiales* sp.

Sampling, genome amplification, sequencing, assembly and tree inference are described in detail elsewhere [36]. Briefly, surface water samples from different stations in Lake Müggelsee (Berlin) were pooled and screened for phytoplankton-infecting parasites. Three host–parasite pairs were isolated by micromanipulation (figure 1 and electronic supplementary material, table S1) and their genomic DNA was amplified with the REPLI-g Advanced DNA Single Cell Kit (Qiagen). Library preparation and whole genome sequencing (PE 150 bp, Illumina NovaSeq) were carried out at Novogene Company Limited (Cambridge, UK). For bioinformatic analyses, the high-performance computing infrastructure at ZEDAT, Freie Universität Berlin, was used [38]. Reads were trimmed and merged using Trimmomatic v. 0.39 [39] and PEAR v. 0.9.11. [40], respectively, and remaining unmerged paired reads were quality filtered with Sickle v. 1.33 [41]. Genomes were then assembled with SPAdes v. 3.15.5 [42] (electronic supplementary material, data) and protein-coding genes were predicted with BUSCO v. 5.1.2 using the database fungi_odb10 [43]. A subset of 265 proteins from two published protein datasets encompassing a broad range of eukaryotic [44] and fungal [31] diversity was chosen according to their presence in the rozellid data and served as query to retrieve homologues from the new data by BLASTp searches [45].

Paralogues and contaminants were removed from the new protein dataset by manual inspection of 265 single-protein maximum likelihood (ML) trees with a high number of taxa (greater than 1000) to facilitate their detection, as described elsewhere [36]. The resulting dataset was reduced to 69 taxa to allow for computationally demanding phylogenomic analyses by removing non-targeted taxa and the fastest-evolving microsporidians (to also prevent long-branch attraction) and by merging taxa into consensus sequences representing OTUs. This taxon-reduced dataset (electronic supplementary material, data) was filtered with PREQUAL v. 1.02 [46], aligned with MAFFT G–INS–I v. 7.475 [47] and further non-homologous residues were removed with Divvier v. 1.01 [48]. Gaps were filtered out (threshold 0.05) with trimAl v. 1.4.1 [49]. The resulting dataset was concatenated into a single matrix using ScaFoS v. 4.42 [50] (265 proteins; 113 816 amino acid positions; electronic supplementary material, data). The newly sequenced Rozellid 19–20 showed a low average data completeness across all proteins of only 3% in the matrix and was therefore removed from the main analysis, resulting in 68 taxa for the final matrix.

Three different phylogenetic inference methods were applied to uncover potential biases introduced by inference methods or evolutionary models: (i) a summary coalescent tree was built with ASTRAL-III v. 5.7.7 [51] using single-protein ML trees inferred with IQ-TREE v. 1.6.12 [52] using best-fitting site-heterogeneous mixture models according to the Bayesian information criterion (BIC; electronic supplementary material, data); (ii) an ML tree was computed with IQ-TREE from the concatenated matrix under the best-fitting site-heterogeneous mixture model LG + C60 + F + R9 and using the posterior mean site frequencies (PMSF) approach [53] with 100 bootstrap replicates; (iii) a Bayesian inference (BI) tree was reconstructed using PhyloBayes-MPI v. 1.8 [54] with the CAT + GTR + G4 model. For BI, three independent Markov chain Monte Carlo (MCMC) chains were run for 2000 generations. The evolution of the log-likelihood at each sampled cycle was monitored and cycles before the stationary phase were removed (burnin = 1000). The three MCMC chains did not show global convergence (maxdiff = 1 and meandiff = 0.0250627).

To reduce systematic biases caused by missing data and to represent the previously excluded Rozellid 19–20 with a reasonable percentage of data, a subset of 100 proteins was selected (85 proteins for which at least one new rozellid sequence was present and 15 proteins with the highest number of taxa; electronic supplementary material, data). This resulted in a protein-reduced matrix with 38 953 amino acid positions and 69 taxa (9% completeness for Rozellid 19–20; electronic supplementary material, table S1), which was analysed by ML as described above.

To phylogenetically relate our new lineages to previously reported rozellid sequences, an ML tree was inferred from a concatenated SSU + LSU rRNA gene alignment. Sequences were extracted from the new genomes with ITSx 1.1.3 [55] and BLASTn searches using genes of described *Rozella* species as query. The same query sequences were used to obtain all publicly available rozellid sequences by BLASTn searches against NCBI's nt database. Sequences were aligned with MAFFT FFT-NS-i and trimmed with trimAl (gap threshold 0.05). An ML tree was inferred from the concatenated alignment using IQ-TREE (GTR + F + R10 model) with ultrafast bootstraps (1000 replicates) [56].

In the new rozellid draft genomes, contigs with potential mitochondrial genes were detected by BLASTn and BLASTp searches against a selection of 266 mitochondrial genomes from a broad diversity of holomycotan linages (excluding Ascomycota and Basidiomycota) available at NCBI. Putative mitochondrion-encoded genes were annotated using MFannot [57]. Nucleotide transporters were searched for by tBLASTn using UniProt's (www.uniprot.org) microsporidian and *Rozella* proteins as queries (PF03219), and taxonomic origin of the predicted ORFs of potential hits was validated by BLASTp searches against NCBI's nr database.

## 3. Results

**Figure 2.**
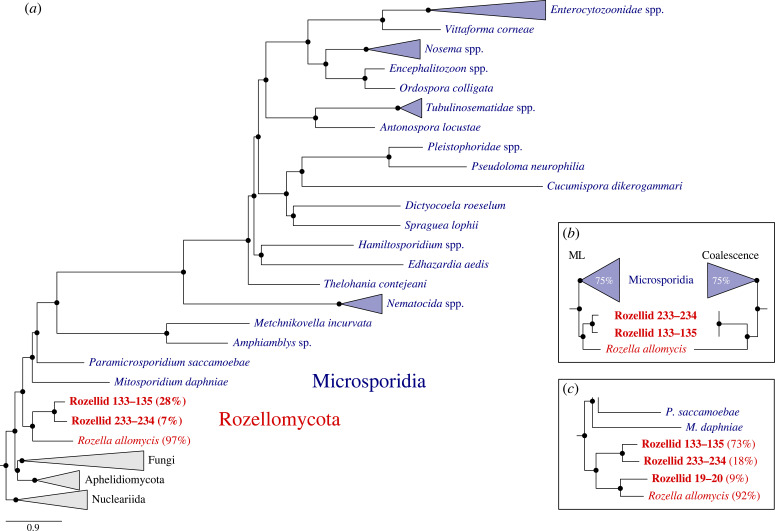
Phylogenomic position of rozellids. (*a*) Bayesian tree inferred under the site-heterogeneous CAT + GTR + G4 model from the full matrix of 113 816 amino acid positions (265 proteins) and 68 taxa. Newly sequenced rozellids are indicated in bold and percentages indicate data completeness in the concatenated alignment. Branches that were fully supported by Bayesian posterior probability are indicated by black circles. (*b*) Maximum likelihood tree inferred under the site-heterogeneous LG + C60 + F + R9-PMSF model from a 113 816 amino acid matrix (left) and coalescence tree inferred under best-fitting (according to BIC) site-heterogeneous models (right). Black circles indicate full branch support (ML, non-parametric bootstrap support from 100 replicates; coalescence, quartet score support without bootstrapping). Percentages in the polygons indicate branch length reductions. (*c*) Detail from an additional ML tree including all three rozellid lineages, which was inferred under the site-heterogeneous LG + C60 + F + R9 model from the reduced matrix with 38 953 amino acid positions (electronic supplementary material, figure S3). Black circles indicate full branch support inferred from ultrafast bootstraps based on 1000 replicates and percentages indicate data completeness in the alignment.

Phylogenomic analyses of new genomic data from three early-branching holomycotan lineages revealed their close relationship to *R. allomycis* (figure 2), supporting a monophyletic rozellid clade that is sister to microsporidians. Congruent rozellid topologies were inferred with maximum support under site-heterogeneous mixture models using both BI (CAT + GTR model; figure 2*a*) and ML (LG + C60 + F + R9-PMSF model, figure 2*b* and electronic supplementary material, figure S1) analyses of a protein matrix with 113 816 sites and 68 taxa and using a coalescence approach (figure 2*b* and electronic supplementary material, figure S2) based on 265 single protein trees (Rozellid 19–20 was inferred with ML only; see below). The BI and ML topologies differed only within the microsporidians, which has been scrutinized before [31]. Average data completeness of the newly sequenced rozellids across all proteins in the concatenated alignment was 7% (Rozellid 233–234) and 28% (Rozellid 133–135; electronic supplementary material, table S1; for reference, *R. allomycis*: 97%). To unravel the phylogenetic position of the third isolated rozellid lineage that showed low data completeness in the alignment (Rozellid 19–20; 3%), we reconstructed an additional ML tree (LG + C60 + F + R9 model) based on a reduced protein sampling to minimize systematic biases caused by missing data (100 proteins; 38 953 sites; 9% data completeness for Rozellid 19–20; electronic supplementary material, table S1) [58]. This tree recovered Rozellid 19–20 to be sister to *R. allomycis*—both forming a well-supported sister group to the two other newly isolated rozellids (figure 2*c* and electronic supplementary material, figure S3). Based on the phylogenetic analysis of the rRNA gene sequences, Rozellids 19–20 and 133–135 were revealed to represent new lineages within the highly diverse Rozellomycota (electronic supplementary material, figure S4). For Rozellid 233–234, SSU/LSU rRNA gene sequences were not detected.

Mitochondrial genes of Complex I were not found in any of the three draft genomes, but a nucleotide phosphate transporter similar to that present in *R. allomycis* [10] was found in one of the three rozellids (Rozellid 133–135; electronic supplementary material, data).

## 4. Discussion

By including new genomic data of uncultured rozellid lineages into a robust phylogenomic framework, we show that the sister relationship of rozellids and microsporidians remained fully supported throughout all analyses, corroborating the previously proposed phylum Rozellomycota [1,8]. This relationship has been inferred before by studying the phylogenetic position of the single rozellid genome of *R. allomycis* with concatenation-based ML and BI analyses [10,31,33,59] but remained controversial in coalescence-based analyses [31,35]. Interestingly, another study [60] on fungal evolution that included additional data of, however, hitherto unpublished rozellid genomes discovered their sister relationship to *M. daphniae* and *P. saccamoebae*—two species, for which we here confirm their proposed assignation to microsporidians [26,31].

The here supported phylogenetic distinction of microsporidians and rozellids corresponds to the microsporidian-specific loss of the flagellum and to the gain of the polar filament, which is involved in spore extrusion when entering the host [25,32] (in *P. saccamoebae* such polar filament is present but inactive [1]). However, the divergence is incongruent with other morphological and cell biological traits that are shared between *R. allomycis* and most but not all microsporidians, such as the loss of the mitochondrial Complex I [25,30,32,61], which is retained in *P. saccamoebae* that has a complete electron transport chain [33]. The sister relationship of microsporidians and rozellids therefore supports the hypothesis of independent losses of Complex I that occurred after their divergence [26,32,33]. Nevertheless, findings of nuclear genes coding for an alternative internal (and external—*R. allomycis* only) NADH dehydrogenase and an alternative oxidase [10,25] suggest that both *R. allomycis* and the early-branching microsporidium *M. daphniae* are capable of producing low amounts of ATP, and are therefore not fully dependent on their hosts' ATP like later-diverging microsporidians that possess more reduced, genome-less mitosomes derived from mitochondria [61]. In this context it is noteworthy that the machinery for nucleotide import, which allows microsporidians to steal their hosts’ ATP [11,62,63], is also present in *R. allomycis* but absent not only in *P. saccamoebae* (with Complex I) but also in *M. daphniae* and metchnikovellids (without Complex I) [10,25,33,34], supporting differential retentions of the ATP transporter acquired via horizontal gene transfer from bacteria in the common ancestor of rozellids and microsporidians [62–64]. In line with these previous findings, NADH dehydrogenase genes (Complex I) were not found in any of our new rozellid draft genomes, but a potential nucleotide phosphate transporter was found in one (Rozellid 133–135). Yet, the fragmented character of the new genomes does not allow final conclusions regarding the hypothesized absence of Complex I or whether our other two rozellids possess a nucleotide transporter. Nevertheless, the documented findings suggest that the here newly presented rozellid lineages, just as *R. allomycis*, produce only low amounts of ATP but compensate for the lack of energy by stealing their hosts' ATP.

Whether our newly isolated rozellids are parasites of phytoplankton or hyperparasites of chytrids that parasitized the phytoplankton cannot be determined from the obtained data. Rozellids were observed to parasitize phytoplankton hosts before [3,12–15], although it was proposed that the separation of the clade containing rozellids and microsporidians from the ancestral holomycotan lineage involved adaptations to an opisthokont host in contrast to its ancestrally phytoplankton-associated sister lineage [65].

In conclusion, we show the phylogenetic sister relationship of rozellids and microsporidians to be robust across all analyses with the inclusion of new genomic data. Our results further corroborate that the evolutionary transition from a functional mitochondrion to a fully reduced mitosome happened independently during early rozellid and microsporidian evolution and that the ancestrally acquired nucleotide transporters as an alternative means to import energy from the host were differentially retained.

## Data Availability

Raw read data are available through NCBI (BioProject no. PRJNA1028182). Additional data (genome assemblies, protein sequences, single-protein trees and matrices) are provided in the electronic supplementary material [66].
